# Supplementary material for: The Caenorhabditis elegans microtubule minus-end binding homolog PTRN-1 stabilizes synapses and neurites
Source: eLife. 2014 Feb 25;3:e01637. doi: 10.7554/eLife.01637 (PMC3930908; doi:10.7554/eLife.01637)
Supplement: Supplementary file 1. — DOI: http://dx.doi.org/10.7554/eLife.01637.015 [file elife01637s001.docx]

Supplemental Table 1 Oligonucleotides used in this study

| Number | Sequence (5’ to 3’) | Purpose |
| --- | --- | --- |
| 583 | CACCGGATTGTCAAACTGC | Genotyping ok364 |
| 586 | CCCAATCGAATCTCCTCCAC | Genotyping ok364 |
| 663 | CGATGAGTCTTGGAATGCAAC | Genotyping ok364 |
| 4216 | GACTCACTTAAGGCGGCCGCGATCTTCAGATGGGA | Constructing NM2498 |
| 4217 | GGAATTCCTGCAGGCTGAAAATAGGGCTA | Constructing NM2498 |
| 4218 | GAATTCCTCGAGATGGCTAGCGTCTCAAAGGGTGAA | Constructing NM2703 |
| 4220 | CTTAAGGCGGCCGCAGTGATTATAGTCTC | Constructing NM2705 |
| 4221 | GGAATTCCTGCAGGCATTTCTAGATGGAT | Constructing NM2705 |
| 4533 | CGATCCTAGGGCATGCCACCGGTGCGTACGAGATCCACTGCCCTTATACAATTCATC | Constructing NM2703 |
| 4586 | CCAGAGATTCCAGCTCGCCCGCAAGT | jsIs1269 analysis |
| 4589 | CCGGAATGAACTGCTCCGTGAGCGCTC | jsIs1269 analysis |
| 4651 | GCACGGCGTACGTAACAAATTTCATATGTT | Constructing NM2704 |
| 4652 | GCACGGCACCGGCGTTAATATTTAAATGTTTC | Constructing NM2704 |
| 4682 | GCAGACGACCTACATTATGTGGGAG | Genotyping ok2415 |
| 4683 | ATGGAACAGTTGGGTGACGTCC | Genotyping ok2415 |
| 4687 | GCTTGCCGGAAATGTATTTATGATTACTTAGT | Genotyping ok2415 |
| 4685 | CTTAACCAACAGCCACAATAATCCACG | Genotyping ok2109 |
| 4686 | TGTCGAGCAAGCCATTGTGGT | Genotyping ok2109 |
| 4741 | CGTAGCCTGCAGGATGGACTTCCCTTTGC | Constructing NM2849 |
| 4742 | CCGACGGCTAGCGTTATTCTTATGAGCCGGAG | Constructing NM2849 |
| 4825 | TCCCTTATTCCAGCCGTCAC | Genotyping tm5597 |
| 4826 | GCTGCCAAAGGGTACGAG | Genotyping tm5597 |
| 4827 | GAGTGTCGCCTTGGGCTTAT | Genotyping tm5597 |
| 4828 | CGATAGGCGGCCGCTACTACTTGTCGCTTGAG | Constructing NM22925 and NM2926 |
| 4829 | CGTAGTCCTGCAGGTGCAAGGACCCCTG | Constructing NM22925 and NM2926 |
| 4931 | CCACAGGGATCTCAAATCGCCC | Genotyping km12 |
| 4932 | CCAGGACATCCGAAATATGTACGAG | Genotyping km12 |
| 4933 | GGCGGATTCGCTCCCTCGC | Genotyping km12 |
| 5031 | CGATGAAGTGGACCAGCCA | Genotyping ok2109 |
